# Robot-assisted enucleation of large dumbbell-shaped esophageal schwannoma: a case report

**DOI:** 10.1186/s12893-018-0370-y

**Published:** 2018-06-05

**Authors:** Yajie Zhang, Yu Han, Jie Xiang, Hecheng Li

**Affiliations:** Department of Thoracic Surgery, Ruijin Hospital, Shanghai Jiaotong University School of Medicine, 197 Ruijin 2nd Road, Shanghai, 200025 China

**Keywords:** Esophageal schwannoma, Robot-assisted, Enucleation

## Abstract

**Background:**

Esophageal schwannomas are extremely rare, with few cases reported in the literature. Traditionally, resection of esophageal schwannoma is typically performed using thoracotomy or video-assisted thoracic surgery. However, large, irregular tumors may increase the surgical difficulties of complete enucleation and lead to potential mucosal damage. Moreover, a subtotal esophagectomy cannot be avoided in some conditions. Here, we report the first case of robot-assisted enucleation of a large dumbbell-shaped esophageal schwannoma.

**Case presentation:**

A 48-year-old woman presenting with a 1-year history of dysphagia was noted to have a homogeneous irregular mass measuring 70 mm in diameter and arising from the submucosal layer of the distal esophagus. A diagnosis of an esophageal submucosal tumor (SMT) was made, most likely leiomyoma. Robot-assisted thoracoscopic excision of the tumor was performed. The da Vinci Surgical System provided delicate dissection in the confined posterior mediastinal space, and the large dumbbell-shaped tumor was completely removed without damage to the mucosal integrity. The operative time was 108 min, and the blood loss was less than 20 ml. The pathology of the tumor was esophageal schwannoma. The patient experienced an unremarkable recovery and was discharged on the fifth day after operation. No symptoms or recurrence were present at the 50-month follow-up postoperatively.

**Conclusion:**

We present a rare case of large irregular esophageal schwannoma that was excised by robot-assisted surgery. A clear operative field and delicate dissections are critical points for the complete removal of this large esophageal SMT. We demonstrate that robotic treatment of large esophageal schwannoma is minimally invasive and can be successfully applied in such cases.

## Background

Esophageal schwannomas are extremely rare and are the least common esophageal submucosal tumors (SMTs), with less than 30 reported cases in the literature [1]. Preoperative differentiation of esophageal schwannomas from other SMTs is difficult, as there are no distinguishing characteristics regarding either symptoms or preoperative imaging tests [2]. A definitive diagnosis is mostly established by pathological examinations after removal of the lesion [3, 4]. Surgical resection is the main treatment for esophageal schwannomas, usually via thoracotomy or video-assisted thoracoscopic surgery (VATS) [5–7]. Here, we report the first case of a large irregular esophageal schwannoma that was successfully removed via robot-assisted thoracoscopic surgery (RATS). The aim of this study is to describe the robot-assisted surgical technique as well as the clinical and pathological features of this unusual tumor.

## Case presentation

**Fig. 1:**
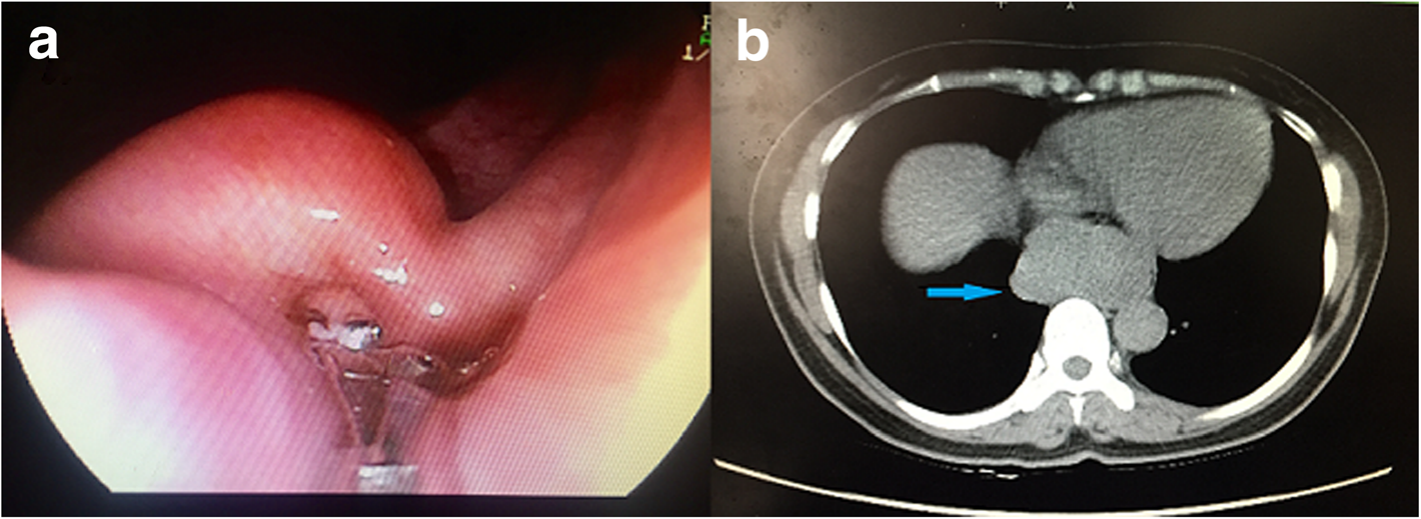
Preoperative imaging of the large esophageal SMT. (**a**) Upper endoscopy revealed a submucosal tumor 70 mm in length that was 30 cm from the incisor with an intact overlying mucosa. (**b**) CT scan of the chest revealed a 69 × 36 mm homogeneous mass in the distal esophageal wall (arrow)

A 48-year-old woman presented to the gastroenterologist at a local hospital with a 1-year history of dysphagia, which had been progressively worsening over the preceding two months. During endoscopy, a bulging tumor 70 mm in length with an intact overlying mucosa was observed in the esophagus 30 cm away from the incisor (Fig. 1a). Endoscopic ultrasonography (EUS) demonstrated a hypoechoic and homogeneous mass in the submucosal layer. Subsequently, a chest computed tomography (CT) scan revealed a homogeneous irregular mass arising from the posterior wall of the distal esophageal wall that was 69 × 36 mm in size and compressed the esophagus and trachea (Fig. 1b). Physical examinations, biochemical tests and cardiopulmonary function were normal. This patient had no medical or family history and was referred to our department for further treatment. Surgical evaluations were discussed preoperatively. Based on the current imaging data, a diagnosis of esophageal SMT was made, most likely leiomyoma. The patient was not recommended further preoperative endoscopic biopsy, which could probably result in mucosal adhesion to the tumor and a subsequently increased risk of mucosal injury during surgery. Taking into consideration the precarious location and large size of the tumor, a decision of robot-assisted resection surgery via the right transthoracic approach was made. The plan was to completely excise the tumor and maintain the integrity of the esophageal mucosa with a low threshold for conversion. Additionally, an esophagectomy with intrathoracic gastroesophagostomy was prepared if the tumor could not be removed.

**Fig. 2:**
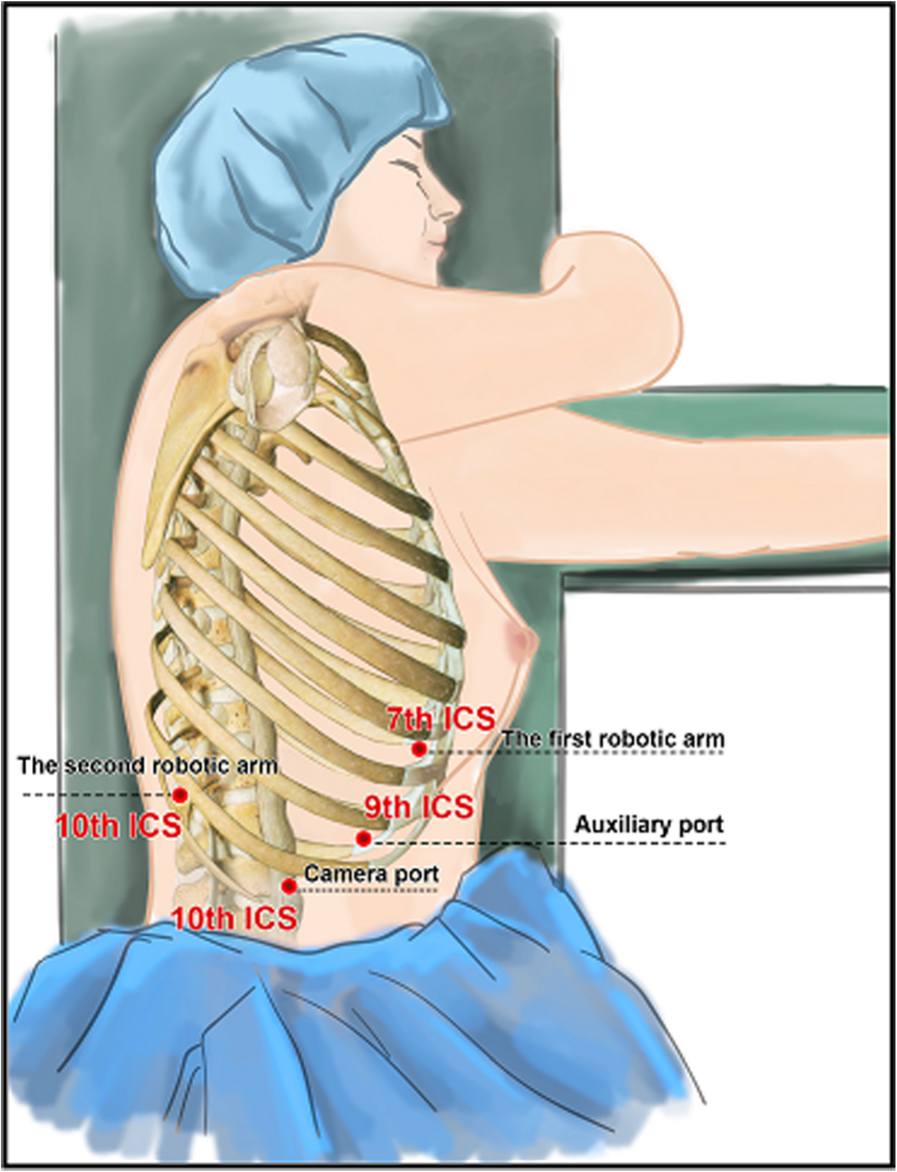
Trocar placement for robot-assisted enucleation of the large esophageal schwannoma

**Fig. 3:**
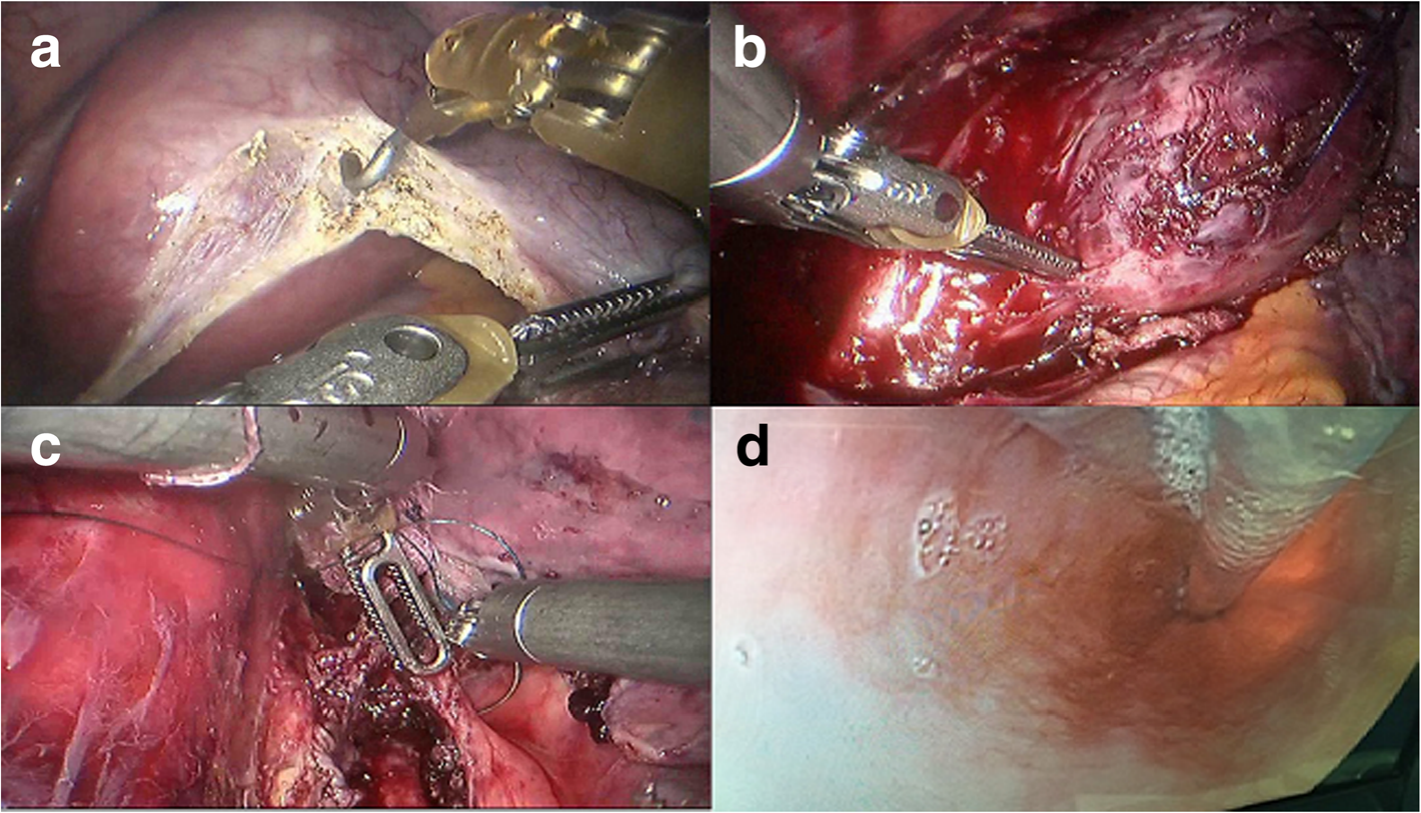
Robot-assisted enucleation of a large esophageal schwannoma. (**a**) By incising the mediastinal pleura, the large tumor was clearly visualized in the distal part of the esophagus. (**b**) The lesion was separated from the surrounding muscle using a combination of sharp and blunt dissection. (**c**) The split muscular layer and mediastinal pleura were loosely reapproximated with 2–0 Vicryl sutures. (**d**) The integrity of the mucosa was confirmed by simultaneous intra-operative upper endoscopy

**Fig. 4:**
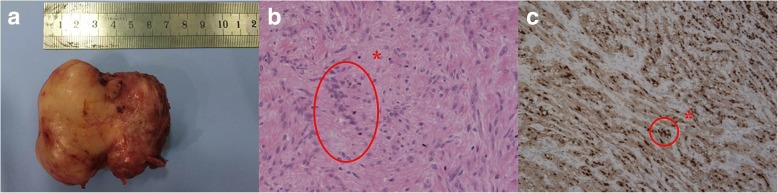
(**a**) The specimen exhibited a well-encapsulated and dumbbell-shaped lesion that measured 70 × 60 × 40 mm. (**b**) Histopathological examination revealed compact bundles of spindle cells (asterisk) (Hematoxylin and eosinstain, original magnification, × 200). (**c**) Immunohistochemical staining showed positivity for S-100 protein (asterisk) (× 100)

The surgical procedure was performed using a da Vinci Surgical System (Model S; Intuitive Surgical, Inc., Sunnyvale, CA, USA). After induction of general anesthesia and placement of a double-lumen endotracheal tube, the patient was placed in the left lateral decubitus position. A 12-mm camera port was inserted at the 10th intercostal space (ICS) at the midaxillary line. Under direct vision, three additional trocars were inserted under thoracoscopic guidance as follows: an 8-mm port in the 7th ICS at the anterior axillary line for the first robotic arm, an 8-mm port in the 10th ICS at the posterior axillary line for the second robotic arm, and finally, a 12-mm auxiliary port in the 9th intercostal space immediately between the camera port and the first robotic port (Fig. 2). We used CO_2_ insufflation with 8–10 mmHg pressure. After all the trocars were positioned, the robot was brought in from the head of the patient with the assistant surgeon on the patient’s left side. The hook or Maryland was manipulated by the first arm of the robot. The Cadiere forceps was manipulated by the second arm. The right lung was retracted laterally, exposing the esophagus. By incising the mediastinal pleura, a large bulge was clearly visualized in the distal esophagus in the confined posterior mediastinal space (Fig. 3a). A longitudinal myotomy was performed to expose the mass, in which a retraction suture was placed by 3–0 Vicryl (Ethicon US, LLC, Cincinnati, OH) and held by an assistant. The lesion was separated from the surrounding muscle and mucosa using a combination of sharp and blunt dissection under three-dimensional vision and wrist-like movement of the robotic instruments followed by enucleation (Fig. 3b). The split muscular layer and mediastinal pleura were loosely reapproximated with 2–0 Vicryl sutures (Fig. 3c). The integrity of the mucosa was confirmed by air insufflation of the esophagus and upper endoscopic inspection (Fig. 3d). The lesion was removed from the thorax in a specimen retrieval bag. Grossly, the 70 × 60 × 40 mm tumor was encapsulated (Fig. 4a). A 32-Fr chest tube was placed into one of the camera port sites under direct vision. The total operative time was 108 min, and the blood loss was less than 20 ml.

The patient was well postoperatively. On the third day, a postoperative gastrograffin swallow demonstrated no leaks or stricture. At this time, the patient started a liquid diet with good tolerance. She was discharged on the fifth day after operation. Histopathological examination revealed compact bundles of spindle cells (Fig. 4b). Immunohistochemical staining was positive for S-100 protein (Fig. 4c). The pathologic diagnosis of the tumor was a benign esophageal schwannoma. The patient remained asymptomatic and exhibited no evidence of recurrence at the 50-month follow-up postoperatively.

## Discussion and conclusions

Esophageal SMTs represent less than 1% of all esophageal neoplasms [8]. Of these lesions, leiomyomas are the most common, accounting for 70–80% [9]. Esophageal schwannomas, which arise from Schwann cells of the neural plexus within the esophageal wall, are the least common esophageal SMTs [10]. Esophageal schwannomas occur frequently in middle-aged women in the proximal esophagus, with lesion dimensions varying from 1 cm to 15 cm [1]. Similar to other esophageal SMTs, including leiomyomas and gastrointestinal stromal tumors (GIST), schwannomas are often asymptomatic. If symptomatic, the most common presenting symptoms are dysphagia and chest discomfort [11]. Confirmation of diagnosis requires pathologic examination with further immunostaining studies after surgical resection. Tumor cells are positive for S-100 protein but negative for smooth muscle markers, such as actin and desmin, which are positive in leiomyoma, and for CD34 and CD117, which are characteristically positive in GIST [2].

The main treatment for esophageal schwannomas is surgical resection [12]. Surgical indications include the presence of symptoms, evidence of an increase in tumor size, and the need to confirm the pathologic diagnosis [2]. Esophageal schwannomas are commonly benign, with only a few reports of malignant cases [13, 14]. Tumor enucleation, as opposed to complete curative resection, is generally sufficient for benign schwannomas [3–5]. The conventional treatment for esophageal schwannomas is transthoracic enucleation through thoracotomy. Currently, VATS is more popular because it is less painful and offers a shorter recovery time than thoracotomy [15]. There are 8 reported cases of esophageal schwannoma resection by VATS in the literature (Table 1). However, the possibility of thoracoscopic enucleation may be limited by the tumor size and location. The total enucleation of large tumors without damage to mucosal integrity is always a challenge due to the limitations of the two-dimensional vision and range of motion of the conventional thoracoscopic instruments. Watanabe et al. reported the difficulty of enucleation of an esophageal schwannoma larger than 5 cm using the VATS approach and the need to convert from enucleation to subtotal esophagectomy [7].

**Table 1:** Literature review of esophageal schwannoma resections by minimally invasive surgery

Case	Author	Year	Age (years)/Sex	Location	Size (mm)	Symptoms	Surgical Approach	Management	Conversion	Operating time (min)	Complications	LOS (d)
1	Chen et al. [6]	2006	73/W	Ut	45 × 50 × 70	Cough, dyspnea, dysphagia	VATS	Enucleation	None	NA	None	NA
2	Mizuguchi et al. [16]	2008	20/W	Ut-Mt	80 × 75 × 60	Dyspnea	VATS	Enucleation	None	NA	None	15
3	Toyama et al. [17]	2008	37/W	Ut	28 × 24 × 19	None	VATS	Enucleation	None	NA	None	4
4	Makino et al. [18]	2013	72/M	Ut	22 × 34 × 29	None	VATS	Enucleation	None	NA	None	NA
5	Shichinohe et al. [19]	2014	61/W	Lt	40 × 30 × 45	Dysphagia	VATS	Enucleation	None	174	None	8
6	Chen et al. [20]	2016	46/M	Mt	30 × 20 × 17/30 × 18 × 15	Discomfort during swallowing	VATS	Enucleation	None	NA	None	5
7	Watanabe et al. [7]	2016	39/W	Ut	39 × 28 × 56	Difficulty swallowing, epigastric pain	VATS	Subtotal esophagectomy	Yes (enucleation to esophagectomy)	NA	None	NA
8	Onodera et al. [21]	2017	47/W	Mt-Lt	60	Dysphagia	VATS	Enucleation	None	498	None	9
9	Our case	2018	48/W	Lt	70 × 60 × 40	Dysphagia	RATS	Enucleation	None	108	None	5

*W,* woman; *M,* man; *Ut*, upper thoracic esophagus; *Mt*, middle thoracic esophagus; *Lt,* lower thoracic esophagus; *VATS*, video-assisted thoracoscopic surgery; *RATS,* robot-assisted thoracoscopic surgery; *LOS*, length of stay

Recently, RATS using the da Vinci Surgical System has provided improved visualization and dexterity in esophageal procedures. In our department, we began to perform robot-assisted esophageal procedures on both malignant and benign tumors in May 2015. In the present case, the tumor was located within the esophageal wall in the confined posterior mediastinum and was 70 mm in diameter. Furthermore, this submucosal mass was irregular with a dumbbell-like shape, which increased the difficulty of obtaining complete enucleation. A decision of transthoracic tumor enucleation using the da Vinci Surgical System was made. The robotic approach offers advantages compared with conventional thoracoscopic systems, including wrist-like movement of the instruments, three-dimensional vision and ergonomic comfort for the surgeon. These features facilitated the combination of sharp and blunt dissection in the confined space and subsequently offered the possibility of complete removal of the tumor without interruption of the capsule and the surrounding esophageal mucosa. The integrity of the mucosa was ensured by the precise dissection with robotic surgery and simultaneous intraoperative upper endoscopy. Moreover, the suture and knot for the repair of the myotomy could be easily performed by the wristed robotic instruments, which prevented mucosal bulging and possible formation of a diverticulum after the enucleation of the large esophageal SMT.

To the best of our knowledge, this is the first report of robot-assisted enucleation of a large esophageal schwannoma. A clear operative field and delicate dissections are key points. This illustrative case demonstrates the utility of robotic surgery in the removal of a large irregular esophageal SMT with respect to its safety and minimal invasiveness.

## Abbreviations

SMT: Submucosal tumor
VATS: Video-assisted thoracoscopic surgery
RATS: Robot-assisted thoracoscopic surgery
EUS: Endoscopic ultrasonography
CT: Computed tomography
ICS: Intercostal space
GIST: Gastrointestinal stromal tumor
LOS: Length of stay

## References

[1] Park BJ, Carrasquillo J, Bains MS, Flores RM (2006). Giant benign esophageal schwannoma requiring esophagectomy. Ann Thorac Surg.

[2] Ha C, Regan J, Cetindag IB, Ali A, Mellinger JD (2015). Benign esophageal tumors. Surg Clin North Am..

[3] Kobayashi N, Kikuchi S, Shimao H, Hiki Y, Kakita A, Mitomi H (2000). Benign esophageal schwannoma: report of a case. Surg Today.

[4] Kitada M, Matsuda Y, Hayashi S, Ishibashi K, Oikawa K, Miyokawa N (2013). Esophageal schwannoma: a case report. World J Surg Oncol.

[5] Tokunaga T, Takeda S, Sumimura J, Maeda H (2007). Esophageal schwannoma: report of a case. Surg Today.

[6] Chen HC, Huang HJ, Wu CY, Lin TS, Fang HY (2006). Esophageal schwannoma with tracheal compression. Thorac Cardiovasc Surg.

[7] Watanabe T, Miyazaki T, Saito H, Yoshida T, Kumakura Y, Honjyo H (2016). Resection of an esophageal schwannoma with thoracoscopic surgery: a case report. Surg Case Rep.

[8] Postlethwait RW, Musser AW (1974). Changes in the esophagus in 1,000 autopsy specimens. J Thorac Cardiovasc Surg.

[9] Seremetis MG, Lyons WS, deGuzman VC, Peabody JW Jr. Leiomyomata of the esophagus. An analysis of 838 cases. Cancer 1976;38:2166–2177. 10.1002/1097-0142(197611)38:5<2166::aid-cncr2820380547>3.0.co;2-b 991129

[10] Kwon MS, Lee SS, Ahn GH (2002). Schwannomas of the gastrointestinal tract: clinicopathological features of 12 cases including a case of esophageal tumor compared with those of gastrointestinal stromal tumors and leiomyomas of the gastrointestinal tract. Pathol Res Pract.

[11] Kassis ES, Bansal S, Perrino C, Walker JP, Hitchcock C, Ross P (2012). Giant asymptomatic primary esophageal schwannoma. Ann Thorac Surg.

[12] Saito R, Kitamura M, Suzuki H, Ogawa J, Sageshima M (2000). Esophageal schwannoma. Ann Thorac Surg.

[13] Mishra B, Madhusudhan KS, Kilambi R, Das P, Pal S, Srivastava DN (2016). Malignant schwannoma of the esophagus: a rare case report. Korean J Thorac Cardiovasc Surg.

[14] Wang S, Zheng J, Ruan Z, Huang H, Yang Z, Zheng J (2011). Long-term survival in a rare case of malignant esophageal schwannoma cured by surgical excision. Ann Thorac Surg.

[15] Luh SP, Liu HP (2006). Video-assisted thoracic surgery--the past, present status and the future. J Zhejiang Univ Sci.

[16] Mizuguchi S, Inoue K, Imagawa A, Kitano Y, Kameyama M, Ueda H (2008). Benign esophageal schwannoma compressing the trachea in pregnancy. Ann Thorac Surg.

[17] Toyama E, Nagai Y, Baba Y, Baba Y, Yoshida N, Hayashi N (2008). A case of thoracoscopically resected benign esophageal schwannoma with high uptake on FDG-PET. Esophagus.

[18] Makino T, Yamasaki M, Takeno A, Kurokawa Y, Miyata H, Takiguchi S (2013). Thoracoscopic enucleation of esophageal schwannoma exhibiting (18) F-fluorodeoxyglucose uptake on positron emission tomography. Dis Esophagus.

[19] Shichinohe T, Kato K, Ebihara Y, Kurashima Y, Tsuchikawa T, Matsumoto J (2014). Thoracoscopic enucleation of esophageal submucosal tumor by prone position under artificial pneumothorax by CO2 insufflation. Surg Laparosc Endosc Percutan Tech.

[20] Chen X, Li Y, Liu X, Fu H, Sun H, Zhang R (2016). A report of three cases of surgical removal of esophageal schwannomas. J Thorac Dis.

[21] Onodera Y, Nakano T, Takeyama D, Maruyama S, Taniyama Y, Sakurai T (2017). Combined thoracoscopic and endoscopic surgery for a large esophageal schwannoma. World J Gastroenterol.

